# Influence of Polyelectrolyte Multilayer Properties on Bacterial Adhesion Capacity

**DOI:** 10.3390/polym8100345

**Published:** 2016-09-26

**Authors:** Davor Kovačević, Rok Pratnekar, Karmen Godič Torkar, Jasmina Salopek, Goran Dražić, Anže Abram, Klemen Bohinc

**Affiliations:** 1 Department of Chemistry, Faculty of Science, University of Zagreb, Zagreb 10000, Croatia; davork@chem.pmf.hr (D.K.); jsalopek@chem.pmf.hr (J.S.); 2 Faculty of Health Sciences, Ljubljana 1000, Slovenia; rokpratnekar@gmail.com (R.P.); karmen.torkar@zf.uni-lj.si (K.G.T.); 3 Jožef Stefan Institute, Ljubljana 1000, Slovenia; goran.drazic@gmail.com (G.D.); anze.abram@ijs.si (A.A.); 4 National Institute of Chemistry, Ljubljana 1000, Slovenia; 5 Jožef Stefan International Postgraduate School, Ljubljana 1000, Slovenia

**Keywords:** bacterial adhesion, *Pseudomonas aeruginosa*, polyelectrolyte multilayers, poly(allylamine hydrochloride), poly(4-styrenesulfonate), scanning electron microscopy, zeta potential

## Abstract

Bacterial adhesion can be controlled by different material surface properties, such as surface charge, on which we concentrate in our study. We use a silica surface on which poly(allylamine hydrochloride)/sodium poly(4-styrenesulfonate) (PAH/PSS) polyelectrolyte multilayers were formed. The corresponding surface roughness and hydrophobicity were determined by atomic force microscopy and tensiometry. The surface charge was examined by the zeta potential measurements of silica particles covered with polyelectrolyte multilayers, whereby ionic strength and polyelectrolyte concentrations significantly influenced the build-up process. For adhesion experiments, we used the bacterium *Pseudomonas aeruginosa*. The extent of adhered bacteria on the surface was determined by scanning electron microscopy. The results showed that the extent of adhered bacteria mostly depends on the type of terminating polyelectrolyte layer, since relatively low differences in surface roughness and hydrophobicity were obtained. In the case of polyelectrolyte multilayers terminating with a positively charged layer, bacterial adhesion was more pronounced than in the case when the polyelectrolyte layer was negatively charged.

## 1. Introduction

Layer-by-layer (LbL) adsorption of polyelectrolytes on various charged surfaces—often known as polyelectrolyte multilayer (PEM) formation—has been intriguing scientists since the early 1990’s, both from fundamental and applicational points of view [1]. Generally, layer-by-layer assembly [2,3] is a versatile method for the fabrication of layered structures from various kinds of component materials (in our case, oppositely-charged polyelectrolytes). Applications are often related to biomedical technologies, but multilayer thin films can also be used in fields such as catalysis, optics, energy, and membranes [4]. Fundamental interest is commonly related to the investigation of linear and/or exponential multilayer growth, and to the effect of experimental conditions (e.g., ionic strength, type of supporting electrolyte, temperature, presence of the anchoring layer) on such build-up processes [5]. In that sense, very important information about the specific behaviour of a certain polyelectrolyte pair could be obtained by examining the corresponding polyelectrolyte complexes [6]. The ease of multilayer formation motivated researchers to extend the type of constituents incorporated into such nanocomposites by including proteins, dendrimers, and even DNA [7]. Therefore, interest in the possible applications of such layered structures has been continuously growing. In that sense, the use of polyelectrolyte multilayered assemblies in biomedical technologies deserves special attention [8]. Recently, the coatings of implantable devices [9,10] and films used for wound healing have been investigated in detail. A special, but very important, case of possible biomedical applications is the adhesion of bacteria on polyelectrolyte multilayers [11,12,13] as a special type of contact surface material. It is known that bacteria readily adhere to wet surfaces, and that they form organized colonies of cells enclosed in a self-excreted matrix composed principally of extracellular polysaccharide (EPS) that facilitates adhesion to the surface and to each other. This type of bacterial organization is termed “biofilm” [14]. Biofilms can be composed of many types of microorganisms, and are irreversibly attached to the surface [15]. In biofilms, microorganisms are protected from stressful/harmful environmental conditions by providing a certain degree of shelter and homeostasis. They are in some kind of competition for and appropriation of available nutrients in a delimited area; on the other hand, they have the benefits of metabolic interactions between microbial species from commensalism, cooperation to mutualism, and gene transfer can be carried out between them for the acquisition of new adaptive phenotypic traits [16,17]. The psychochemical properties of cell surfaces are an important aspect in active bacterial adhesion to different material surfaces. The surfaces of most bacterial cells are negatively charged [18]. Such bacteria feel a repulsive force close to a negatively-charged surface, which keeps them at a short distance away. However, the bacterial cell-surface possesses fimbriae, flagella, and a lipopolysaccharide (LPS) layer in the cell wall of Gram-negative bacteria. The main function of fimbriae is to overcome the initial electrostatic repulsion barrier that exists between the cell and the substratum [19]. The hydrophilic LPS layer reduces the cell’s ability to interact with hydrophilic surfaces [20]. The basic stages of bacterial adhesion are generally described by a two-stage kinetic binding model [21]: an initial, rapid, and easily reversible interaction between the bacteria cell surface and the material surface, followed by a second stage that includes specific and nonspecific interactions between so-called adhesion proteins expressed on bacterial surface structures (fimbriae or pili) and binding molecules on the material surfaces. The factors that affect the intensity of bacterial adhesion include material surface roughness, charge, degree of hydrophobicity, Lewis acid–base character, and hydrogen-bonding capacity [15,21,22]. Environmental factors, including pH, temperature, nutrient composition, and population characteristics may enhance the adhesion and biofilm maturation [23].

To estimate the number of bacteria on the surfaces, it is necessary to understand the limitations of different direct count techniques. The average size of a bacterial cell is around 1 μm, which is close to the resolution limit of optical methods such as epifluorescence microscopic count. Scanning electron microscopy offers higher resolution and superior depth of focus. Because imaging is in vacuum, great care should be taken during the hydrated sample preparation. Using a backscattered electron detector for image formation is especially useful, since those high-energy electrons generate an image that is related to the average atomic number of the sample (Z contrast). Unfortunately, both the bacteria and the polymer coating used in this study are carbon-based, so no contrast difference could be expected. However, topological information gathered by secondary electrons gave sufficient contrast for simple bacteria counting [24,25].

Taking into account that PEM-modified surfaces exhibit significantly improved bacterial anti-adhesive properties, in this paper we decided to relate the conditions (polyelectrolyte concentration, salt concentration) at which the polyelectrolyte multilayers are formed, the number of polyelectrolyte layers, and the type of the terminating layer with corresponding bacterial adhesion. For that purpose, we applied zeta-potential measurements to determine the charge of the polyelectrolyte multilayers formed on silica particles, and scanning electron microscopy to monitor the adhesion of bacteria. As oppositely-charged polyelectrolytes, we used poly(allylamine hydrochloride) (PAH) and sodium poly(4-styrenesulfonate) (PSS). These synthetic polyelectrolytes have been widely used in the process of polyelectrolyte multilayer formation, and we also used them extensively in our investigations of polyelectrolyte complexes [6]. In order to examine the adhesion of bacteria on PAH/PSS multilayers, we used *Pseudomonas aeruginosa* (*P. aeruginosa*), a common pathogenic bacterium with a possible multidrug resistance by mutation which is often responsible for postoperative infections. It was shown recently [26] that clinically-relevant pathogenic strains such as *P. aeruginosa* could be inactivated, for example, by photocatalytically-active titanium dioxide functionalized with silver nanoparticles and immobilized in a polyacrylate-based nanohybrid thin film.

## 2. Materials and Methods

### 2.1. Materials

#### 2.1.1. Polyelectrolytes

The cationic polyelectrolyte poly(allylamine hydrochloride) (PAH) *M*_w_ = 15,000 g·mol^−1^, and the anionic polyelectrolyte sodium poly(4-styrenesulfonate) (PSS) *M*_w_ = 77,000 g·mol^−1^ were purchased from Aldrich (St. Louis, MO, USA). The salt NaClO_4_ was obtained from Sigma Aldrich (St. Louis, MO, USA), and was of analytical purity grade.

#### 2.1.2. Substrates

To monitor the adhesion of bacteria, we used silicon wafers carrying an oxide (silica) layer of about 70 nm (produced by Wafernet, Inc., San Jose, CA, USA) as the solid substrate, while for the zeta potential measurements, silica particles (Aerosil 200, Degussa, Frankfurt am Main, Germany) were used as the solid substrate.

#### 2.1.3. Bacteria

In our experiment, a standard strain *P. aeruginosa* ATCC 27853 (Czech Collection of Microorganisms, Brno, Czech Republic), isolated from blood culture was used. *P. aeruginosa* is an aerobic Gram-negative bacterium with some polar flagella. It is a slightly curved rod with the length of 1 to 5 µm and a diameter between 0.5 and 1 µm [27], and is commonly found in the environment (e.g., soil, water, and other moist locations). *P. aeruginosa* can cause pneumonia, ulcerative keratitis, inflammation of the skin, and soft tissue, urinary tract, and postoperative infections. Bacteria from the collection were transferred on nutrient agar. After incubation at 37 °C for 24 h, a single colony of the strain was transferred from nutrient agar to the nutrient broth without glucose (Biolife, Bolzano, Italy) and incubated at the same conditions. The surface charge of the bacteria was determined earlier [18] by measuring the zeta potential of *P. aeruginosa* in two phosphate buffer solutions at two ionic strengths (1 and 100 mmol/L). The results showed negative zeta potentials: −16.92 ± 2.42 mV for 1 mmol/L and −7.85 ± 12.8 mV for 100 mmol/L.

### 2.2. Polyelectrolyte Multilayer Preparation

#### 2.2.1. Multilayers on Silica Particles

Polyelectrolyte multilayers on silica particles used for the determination of zeta potential were prepared as follows: In step 1, silica particles were suspended in PAH solution containing an appropriate amount of NaClO_4_. The system was mixed for 10 min, centrifuged, rinsed with NaClO_4_ solution, and mixed again for 10 min. After rinsing, the sample for zeta potential measurements was transferred into the cuvette, suspended using ultrasound, and measured. In step 2, the silica particles with the adsorbed PAH layer were suspended in PSS solution containing an appropriate amount of NaClO_4_. The rest of the procedure in step 2 was the same as in step 1. The same holds true for the other added polyelectrolyte layers.

Since poly(allylamine) hydrochloride is a weak polycation, and poly(4-styrenesulfonate) is a strong polyanion, both polyelectrolyte solutions were prepared in 1 × 10^−4^ mol·dm^−3^ hydrochloric acid in order to obtain the pH of both solutions to be ~4. It was shown earlier [28] that the multilayer thickness is independent of the chain length when fully charged PAH (at pH ~ 4) is combined with fully charged PSS. At that pH, silica particles are still negatively charged, since the isoelectric point of silica is at pH ~ 3.

#### 2.2.2. Multilayers on Silica Plates

Polyelectrolyte multilayers for monitoring the adhesion of bacteria were prepared by adsorbing the polyelectrolytes on a silica surface, as described by Decher [1]. The concentration of both polyelectrolyte solutions (PAH and PSS) was 0.01 mol·dm^−3^. In the beginning, we rinsed substrate plates in NaClO_4_ solution (*c* = 0.001 mol·dm^−3^) for 5 min. After that, the adsorption process started by dipping substrate plates into a solution of a positively-charged polyelectrolyte (PAH). After 10 min of dipping, we rinsed the plates with salt solution again. With that procedure, we flushed unbound positive polyelectrolytes from the surface. Following this, the adsorption of the second layer was performed by dipping the SiO_2_ plates with adsorbed polycation layer into a solution of the negatively charged polyelectrolyte (PSS) for 10 min. After 10 min, we transferred those plates into a solution of NaClO_4_ for another 5 min to flush unbound negative polyelectrolytes from the surface. We repeated the procedure of adsorbing layers until we obtained a plate that was covered with five layers having positively charged polyelectrolyte (PAH) as the terminating layer, as in the case of zeta potential measurements. Moreover, in order to examine the influence of the charge of the terminating layer, we also prepared a plate that was covered with six layers having negatively charged polyelectrolyte (PSS) as the terminating layer.

### 2.3. Surface Characterization

#### 2.3.1. Electrokinetic Measurements

The electrophoretic mobilities (μ) of pure silica particles and of silica particles covered with polyelectrolytes were determined by means of a ZetaPlus instrument (Brookhaven Instruments Corporation, Brookhaven, NY, USA) at 25 °C from the measured Doppler shift in angular frequency (Δω) and the applied electric field (*E*)

(1) $$\Delta \omega =\left(\frac{2\pi n}{\lambda }\right)E\;\mu \sin\theta$$

where *n* is the medium refractive index and λ the incident wavelength. The scattering angle during measurement was constant (θ = 15°). The zeta potential (ζ) was calculated from measured electrophoretic mobility by Smoluchowski equation

(2) $$\zeta =\frac{\mu \eta }{\varepsilon_{0}\varepsilon_{r}}$$

where ε_r_ is the relative permittivity, ε_0_ the electric permittivity of vacuum, and η is the viscosity.

#### 2.3.2. Surface Roughness Measurements

Atomic force microscopy (AFM, VEECO Dimension 3100 system in contact mode, Veeco, New York, NY, USA) was used for characterization of the surface topography of the substrate (oxidized silicon wafer with adsorbed polyelectrolyte multilayers) on a nanometer scale. This method has the possibility of imaging surfaces with high resolution and the quantitative evaluation of selected surface features, including statistical analysis, which permits the roughness parameters to be determined.

#### 2.3.3. Surface Hydrophobicity Measurements

A Theta Optical Tensiometer (Attension, Stockholm, Sweden) was used for the determination of the contact angle between a drop of water and the substrate (oxidized silicon wafer with adsorbed polyelectrolyte multilayers). The tensiometer consists of a light source, a camera, a liquid dispenser, and a sample stage where the substrate is placed. A small drop of water was placed on the substrate using a dispenser, and an image of the profile of the drop was taken by means of a digital camera and a light source at opposite side of the drop. The angle between the substrate and the drop is determined from the image. The contact angle is a measure for the surface hydrophobicity.

### 2.4. Monitoring of the Bacterial Adhesion on Surfaces

We sterilized plates with adsorbed polyelectrolytes with UV light (*λ* = 254 nm, 30 W) and then transferred them into *P. aeruginosa* culture, obtained by dilution (1:300) of the overnight culture, containing approximately 10^7^ CFU mL^−1^. After 18 h of incubation at 37 °C in aerobic conditions, we removed the plates from the culture broth and rinsed them five times with 5 mL of 0.1 mol·dm^−3^ sterile PBS buffer, once with 3 mL of sterile distilled water, and dried them for 10 min with hot air at 60 °C to fix them.

With a GATAN Model 682 PECS system (Precision Ion Etching and Coating System, GATAN Inc., Pleasanton, CA, USA), we applied a thin layer of gold (7 nm) on the plates with adsorbed PEM surfaces. We qualitatively and quantitatively analysed plates on the scanning electron microscope (Jeol JSM-7600F, Jeol, Tokyo, Japan). With qualitative analysis, we made visual assessment of bacterial adhesion on polyelectrolyte multilayers terminating with a polycation and a polyanion layer, respectively. For quantitative analysis, we first manually encircled bacteria on SEM images and converted images to binary form. For editing and measuring the fraction of the surface covered with bacteria, we used ImageJ software package (Version 1.50b, 2015, Wayne Rasband, National Institutes of Health, Bethesda, MD, USA). Several images, representing a total area of 500 µm^2^, taken at different sample positions, were analysed. In this way, we got coverage of *P. aeruginosa* bacteria on terminating polycation and polyanion layers and compared the results.

## 3. Results

### 3.1. Zeta Potential of the SiO_2_ Particles Covered with PEMs

The zeta potential measurements could be divided in two groups, depending on the aims that we wanted to achieve. In the first part, the zeta potential values obtained using various polyelectrolyte concentrations are presented, and in the second part, we investigated the influence of added supporting electrolyte concentration on the formation of polyelectrolyte multilayers. Sodium perchlorate was chosen as the added electrolyte.

**Figure 1:**
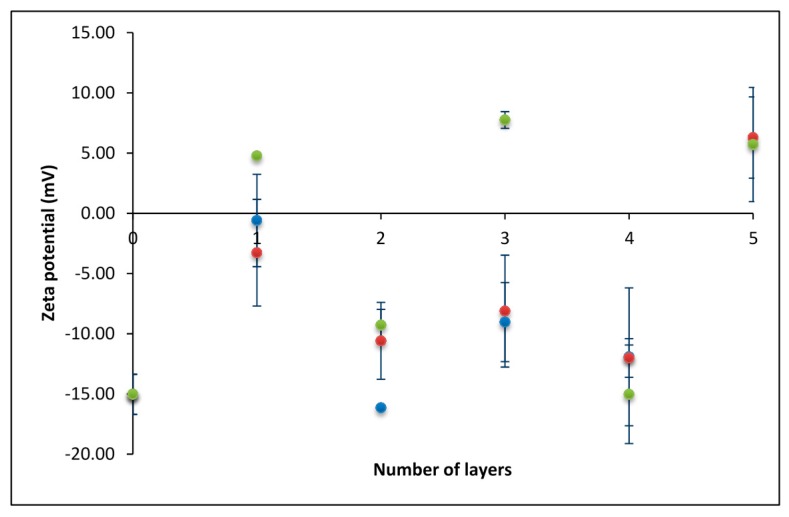
The dependence of zeta-potential on the number of polyelectrolyte layers obtained at three different polyelectrolyte concentrations. *c*_(PAH)_ = *c*_(PSS)_ = 0.001 mol·dm^−3^ (**blue dots**); *c*_(PAH)_ = *c*_(PSS)_ = 0.003 mol·dm^−3^ (**red dots**); and *c*_(PAH)_ = *c*_(PSS)_ = 0.01 mol·dm^−3^ (**green dots**). PAH: poly(allylamine hydrochloride); PSS: sodium poly(4-styrenesulfonate).

For the first experiment, we used relatively low monomer concentrations (*c*) of polyelectrolytes (*c*_(PAH)_ = 0.001 mol·dm^−3^, *c*_(PSS)_ = 0.001 mol·dm^−3^). As shown in Figure 1 (blue dots), the polyelectrolyte concentrations used were not high enough to obtain the systematic overcharging of the sample. The charge of the silica particles covered with both polyelectrolytes was always negative: more negative after the adsorption of PSS, and less negative after the adsorption of PAH. The exception was the last (fifth) layer, which gave a slightly positive zeta potential. In the next experiment (Figure 1, red dots), we increased the concentrations of polyelectrolytes to *c*_(PAH)_ = 0.003 mol·dm^−3^ and *c*_(PSS)_ = 0.003 mol·dm^−3^. However, that relatively slight increase did not lead to the systematic overcharging of the system. Finally, in the third experiment (green dots), concentrations were additionally increased to 10 times higher than in experiment 1 (*c*_(PAH)_ = 0.01 mol·dm^−3^, *c*_(PSS)_ = 0.01 mol·dm^−3^) and in this case, overcharging was obtained. In all experiments presented in Figure 1, the concentration of NaClO_4_ solution was kept constant (*c*_(NaClO4)_ = 0.01 mol·dm^−3^).

**Figure 2:**
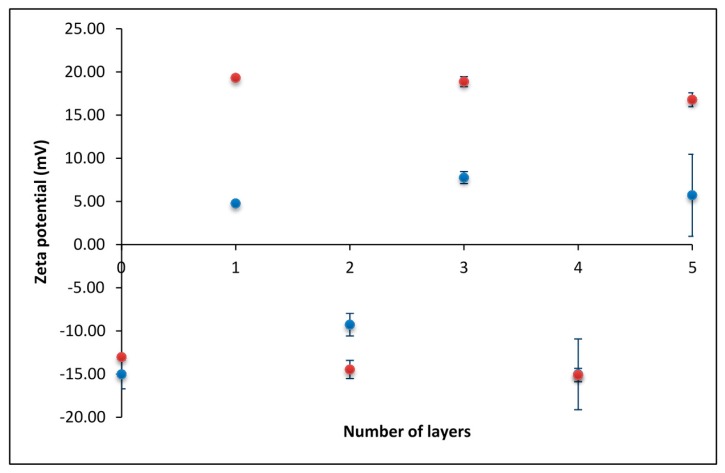
The dependence of zeta potential on the number of polyelectrolyte layers obtained at two different salt (NaClO_4_) concentrations: *c* = 0.001 mol·dm^−3^ (**red dots**); and *c* = 0.01 mol·dm^−3^ (**blue dots**).

After determining the concentration of polyelectrolytes needed for overcharging to take place, we investigated the effect of supporting electrolyte concentration on polyelectrolyte multilayer formation. In Figure 2, the results obtained in the presence of two NaClO_4_ concentrations are compared: *c*_(NaClO4)_ = 0.001 mol·dm^−3^ (red dots) and *c*_(NaClO4)_ = 0.01 mol·dm^−3^ (blue dots). As expected, at higher salt concentrations, the absolute values of the obtained zeta potentials were lower, which could be attributed to the more pronounced screening of polyelectrolyte charges.

### 3.2. Adhesion of Bacteria on Polyelectrolyte Multilayers

**Figure 3:**
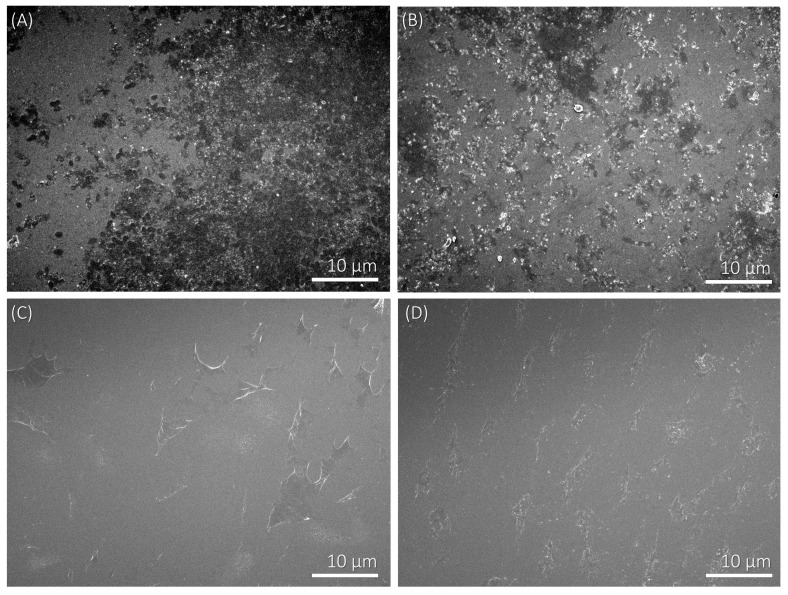
SEM micrographs of *P. aeruginosa* bacteria adhered on the polyelectrolyte multilayer surface at 2000× magnification. (**A**) Adhered bacteria on a SiO_2_ plate which is covered with five polyelectrolyte layers and the terminating layer has a positive electric charge (PAH); (**B**) Adhered bacteria on a SiO_2_ plate which is covered with six layers and the last adsorbed layer has a negative electric charge (PSS); (**C**) The negative control of SiO_2_ plates with five adsorbed polyelectrolyte layers; (**D**) The negative control of SiO_2_ plates with six adsorbed polyelectrolyte layers.

**Figure 4:**
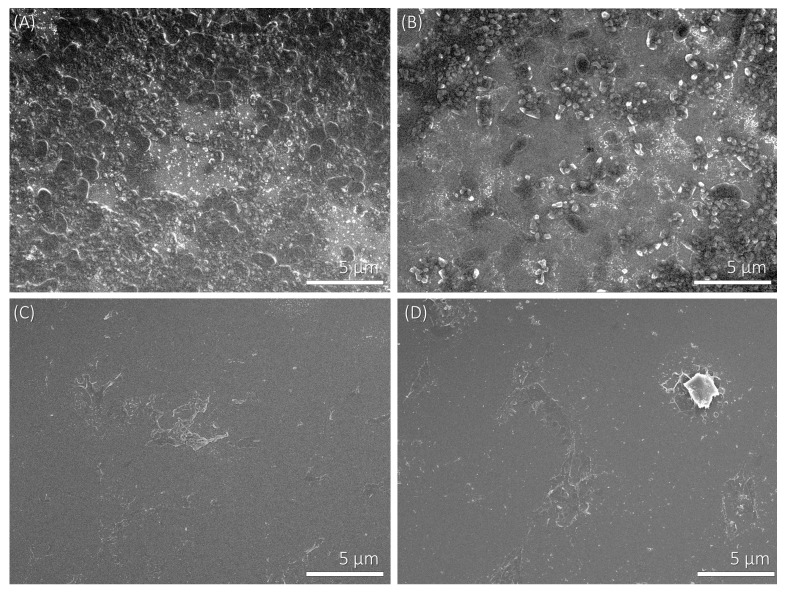
SEM micrographs of *P. aeruginosa* bacteria adhered on the polyelectrolyte multilayer surface at 5000× magnification. (**A**) Adhered bacteria on a SiO_2_ plate which is covered with five polyelectrolyte layers and the terminating layer has a positive electric charge (PAH); (**B**) Adhered bacteria on a SiO_2_ plate which is covered with six layers and the last adsorbed layer has a negative electric charge (PSS); (**C**) The negative control of SiO_2_ plates with five adsorbed polyelectrolyte layers; (**D**) The negative control of SiO_2_ plates with six adsorbed polyelectrolyte layers.

**Figure 5:**
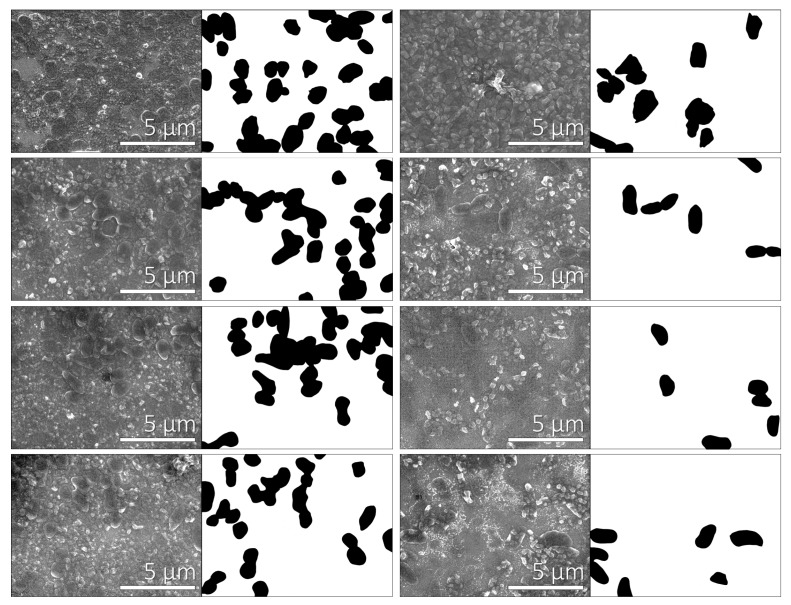
Qualitative analysis of SEM images at 10000× magnification. Column 1: Bacteria adhered on the positive polyelectrolyte layer (SiO_2_ plates with five polyelectrolyte layers); column 2: Black and white pictures of adherent bacteria on SiO_2_ plates that were covered with five layers. The first picture had 31.4% of the surface covered with bacteria, the second 25.3%, the third 25.9%, and the fourth picture 19.5%; column 3: Adherent bacteria on the negative polyelectrolyte layer (SiO_2_ plates with six polyelectrolyte layers); column 4: Black and white pictures of adherent bacteria on SiO_2_ plates that were covered with five PEMs. The first picture had 14% of the surface covered with bacteria, the second 6.1%, the third 6.4%, and the fourth picture 7.3%.

For studying the adhesion of bacteria on polyelectrolyte multilayers, we applied the procedure described in Section 2.2.2. The experimental conditions for multilayer formation were chosen on the basis of the zeta potential results presented in Figure 1 and Figure 2. Therefore, the concentration of both positively and negatively charged polyelectrolytes was 0.01 mol·dm^−3^, and the added supporting electrolyte (NaClO_4_) concentration was 0.001 mol·dm^−3^. We investigated two cases; i.e., polyelectrolyte multilayers that contained (a) PAH and (b) PSS as the terminating layer. The levels of bacterial adhesion on polyelectrolyte multilayers terminating with a PAH or a PSS layer were determined from the SEM images (Figure 3, Figure 4 and Figure 5). Bacterial adhesion was more pronounced on the plates with five adsorbed polyelectrolyte layers; i.e., in the case where the last adsorbed layer had a positive electric charge (adsorbed cationic polymer—PAH). On such a positively-charged surface, many different colonies of adhered *P. aeruginosa* bacteria were observed. Lower adhesion was observed on the plates with six adsorbed polyelectrolyte layers, where the last adsorbed layer was negatively-charged (adsorbed anionic polymer—PSS). In SEM pictures, grey areas are the multilayer surface, whereas black dots are adhered bacteria on the surface and white areas are salt crystals.

The fraction of the surface covered with bacteria was determined to be 20.4% ± 4.8% on the plates with five adsorbed layers (where the terminating layer was positively charged). On the other hand, on the plates with six adsorbed layers (where the terminating layer was negatively charged), only 9.0% ± 3.1% of the surface was covered with bacteria. We calculated the fraction of bacterial adhesion on the terminating polycation layer (five layers) from 22 SEM images, and on the terminating polyanion layer (six layers) from 15 SEM images.

## 4. Discussion

In this article, we analyse the impact of surface charge density on bacterial adhesion potential. We have chosen silica surfaces on which PAH/PSS polyelectrolyte multilayers were formed. The surface charge density was determined by zeta potential measurements of silica particles covered with polyelectrolyte multilayers. The surface roughness and hydrophobicity were measured with AFM and tensiometer, respectively. These measurements served as control for the effects which could potentially influence the adhesion extent. Finally, the extent of bacterial adhesion was determined using SEM.

**Scheme 1:**
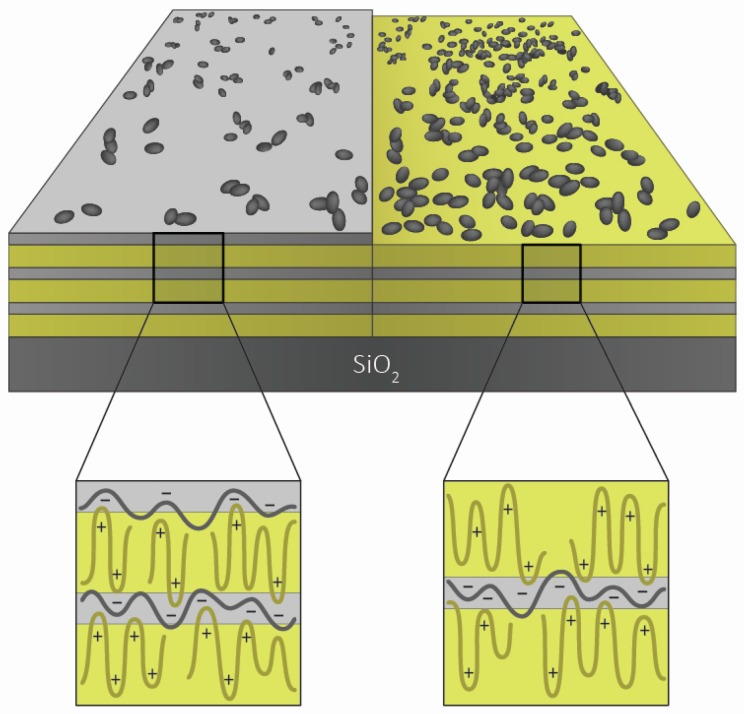
Schematic presentation of PAH/PSS polyelectrolyte multilayers terminating with PSS (6 layers) and PAH (5 layers).

It is known [29] that the substrate to which the bacteria adhere should be well characterized, especially in terms of its charge, hydrophobicity, and roughness. That is why in the first part of the presented study, we investigated the charge of the silica particles covered with polyelectrolyte layers. The idea was to examine the experimental conditions for the preparation of the regular polyelectrolyte multilayer formed from PAH and PSS. Therefore, we conducted the zeta potential measurements in order to establish which polyelectrolyte concentrations and ionic strengths are optimal for the PEM build-up. It was shown that a certain critical polyelectrolyte concentration is needed for the multilayers to exhibit the change from negative to positive surface charge during the process of alternate adsorption of oppositely-charged polyelectrolytes (Figure 1). The effect of ionic strength was tested using the example of added sodium perchlorate, whose influence on polyelectrolyte complexation was extensively examined in our previous studies [6,30]. As expected, the increase in salt concentration led to a decrease in the absolute value of zeta potential (Figure 2). The schematic presentation of the obtained polyelectrolyte multilayers is presented in Scheme 1. On the basis of these results, the experimental conditions for the preparation of PEMs to be used for testing the adhesion of bacteria were determined.

As stated earlier, among the key factors that influence the process of bacterial adhesion, the importance of the charge, the material surface roughness, and the degree of hydrophobicity should be stressed. In the experiments shown here, the adhesion of bacteria was studied on the polyelectrolyte multilayers terminating with two different polyelectrolytes: a polycation and a polyanion. Therefore, not only the electric charge, but also other surface properties could have a significant impact on adhesion [12]. For this reason, we also examined the surface hydrophobicity and the surface roughness of polyelectrolyte multilayers formed on oxidized silicon wafers prior to bacterial adhesion. The measured hydrophobicity (i.e., contact angle) was similar on polyelectrolyte multilayers terminating with a polycation and with a polyanion layer. In the first case, the contact angle was 48.9° ± 2.5°, and in the latter, 46.9° ± 5.0°.

The results of roughness obtained by means of atomic force microscopy were also similar for both types of multilayers. In the case of the positive surface (polycation terminating multilayer), roughness was determined to be 0.017 ± 0.004 µm, and in the case of the negative surface (polyanion terminating multilayer), it was 0.019 ± 0.006 µm.

All the results are systematized in Table 1. Such relatively low differences in roughness and hydrophobicity should not cause significant differences in the level of bacterial adhesion between both layers. These results allow us to assume that the differences in bacterial adhesion capability between the systems with oppositely charged terminating layers should most probably be the result of electrostatic interactions. The adhesion results presented in Figure 3, Figure 4 and Figure 5 are therefore coherent with predictions if we take into account that bacterial cell walls possess negative charges (Bohinc et al. [21]).

In the past, the influence of electrostatic interactions on bacterial adhesion to various surfaces has been studied. Like-charged surfaces repel in the presence of monovalent ions [31]. Increased ionic strength showed increased deposition of negatively charged bacteria on the negatively charged material surfaces [32]. Contrary to like-charged surfaces, the surfaces of opposite charge attract. It was shown that the positively-charged domains at the bacterial surface yield an electric double layer attraction with a negatively charged material surface [33,34].

Generally, there are two types of driving forces which influence the adhesion of bacteria to material surfaces. These are the forces between bacteria and the surface in its vertical direction, and the lateral forces between neighbouring bacteria. The first determines the amount of bacteria which come in contact with the surfaces, whereas the latter determines the possible aggregation between bacteria. In our case, both forces are present, leading to bacterial adhesion on the polyelectrolyte multilayer surface, as well as to bacterial aggregation. The interplay between these two types of forces leads to the obtained results.

## 5. Conclusions

There is no doubt that polyelectrolyte multilayers are surfaces of great interest for possible applications in biomedical technologies [35], and especially for the adhesion of bacteria. However, special care should be taken in the process of forming PEMs in order to control the possible adhesion. We showed here that both polyelectrolyte and supporting electrolyte (added salt) concentrations significantly influence the multilayer build-up. Therefore, in the process of polyelectrolyte multilayer formation, special attention should be given to these experimental parameters. Of course, other experimental conditions which are known to influence the PEM build-up, such as pH, type of added supporting electrolyte, molecular weight, and temperature should also be taken into account. Moreover, the surface properties, such as surface roughness and hydrophobicity should also be carefully examined, as well as the bacterial properties. In the future, we plan to investigate the interactions between particular bacteria and material surface, as we measured the force between borosilicate glass and Gram-positive bacteria earlier [36]. The proteins (e.g., lysozyme) will be also included in the process of multilayer build-up.

This study has shown the importance of combining surface characterization with microbial testing to understand the bacteria–surface interactions. The application of polyelectrolyte multilayers with tunable electrostatic properties enables the preparation of the surfaces with desirable properties, in terms of bacterial adhesion.

## Figures and Tables

**Table 1:** Characterisation of PAH/PSS polyelectrolyte multilayers with PAH or PSS as the terminating layer in terms of terminating layer charge (i.e., zeta potential), contact angle value, roughness, and finally the fraction of the corresponding multilayer covered with bacteria.

**Quantity**	PAH as terminating layer (five layers)	PSS as terminating layer (six layers)
**Zeta potential**	16.78 mV	−15.11 mV
**Contact angle**	48.9° ± 2.5°	46.9° ± 5.0°
**Roughness**	0.017 ± 0.004 µm	0.019 ± 0.006 µm
**Fraction of the multilayer surface covered with *P. aeruginosa***	20.4% ± 4.8%	9.0% ± 3.1%
